# Research Review: Evaluating and reformulating the developmental taxonomic theory of antisocial behaviour

**DOI:** 10.1111/jcpp.12102

**Published:** 2013-07-04

**Authors:** Graeme Fairchild, Stephanie HM Goozen, Andrew J Calder, Ian M Goodyer

**Affiliations:** 1 Academic Unit of Psychology, University of SouthamptonSouthampton, UK; 2 School of Psychology, Cardiff UniversityCardiff, UK; 3 Medical Research Council Cognition and Brain Sciences UnitCambridge, UK; 4 Department of Psychiatry, University of CambridgeCambridge, UK

**Keywords:** Antisocial behaviour, conduct disorder, developmental taxonomic theory, epidemiology, neuropsychology, neuroimaging

## Abstract

**Background**The developmental taxonomic theory proposes that there are two subtypes of antisocial behaviour. The first is a neurodevelopmental disorder which emerges in early childhood and follows a life-course persistent course, whereas the second emerges in adolescence, remits in early adulthood and reflects peer processes such as mimicry of antisocial peers. The aim of this review was to evaluate the developmental taxonomic theory in the light of recent empirical research.

**Methods**We conducted a comprehensive literature review comparing these subtypes of antisocial behaviour based on searches on PubMed and other scientific databases covering the period from 1993 to 2013. We focused on research encompassing psychiatric epidemiology, personality assessment, neuropsychology, neuroendocrinology, genetics, and structural and functional neuroimaging. Sixty one empirical studies were identified that investigated one of these forms of antisocial behaviour separately or explicitly compared childhood-onset and adolescence-onset forms of antisocial behaviour.

**Results**Empirical research provides support for the hypothesis that life-course persistent antisocial behaviour is a neurodevelopmental disorder which emerges in the transactions between individual vulnerabilities and environmental adversity. In contrast to the developmental taxonomic theory, however, empirical findings suggest that severe antisocial behaviour that emerges in adolescence frequently has a negative prognosis and is rarely limited to the adolescent period. In addition, both forms of antisocial behaviour are associated with emotion processing deficits, changes in brain structure and function, alterations in cortisol secretion, and atypical personality traits (such as increased callous-unemotional traits).

**Conclusions**We conclude that the developmental taxonomic theory is in need of revision, as differences between life-course persistent and adolescence-onset forms of antisocial behaviour appear to be quantitative, rather than qualitative, in nature. In addition, evidence is accumulating that adolescence-onset antisocial behaviour may also be a neurodevelopmental disorder. To account for the similarities between these groups, despite the differences in their age-of-onset, we propose that the quality of the child's early environment moderates the relationship between individual vulnerabilities and the age-of-onset of antisocial behaviour.

## The developmental taxonomic theory and its impact on developmental psychology and psychopathology

**Figure 1:**
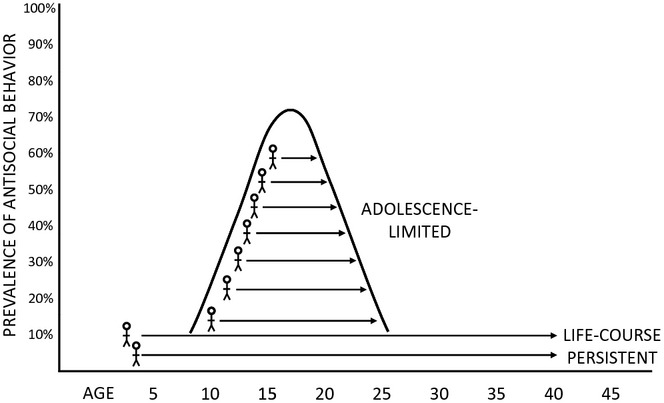
A schematic representation of the developmental taxonomic theory of antisocial behaviour, illustrating the hypothetical courses of life-course persistent (LCP) and adolescence-limited (AL) forms of antisocial behaviour. (Adapted with permission from Moffitt, 1993, *Psychological Review*, 100, p. 677; Copyright American Psychological Association, 1993.)

The developmental taxonomic theory was first described in a seminal and highly influential article published by Terrie Moffitt in 1993 (Moffitt, 1993). It holds that within adolescent populations, there are two groups of offenders who differ systematically in the courses, correlates and causes of their antisocial behaviour: a life-course persistent (LCP) group who show stable high levels of aggression and antisocial behaviour starting in childhood and continuing into adulthood, and who are characterised by neuropsychological impairments and exposure to childhood adversity, and an adolescence-limited (AL) group whose antisocial behaviours are primarily nonaggressive, and who do not show neuropsychological impairments (see Figure 1). In fact, Moffitt explicitly excluded a role for neuropsychological deficits or neurobiological factors in the aetiology of AL antisocial behaviour: ‘Instead of a biological basis in the nervous system, the origins of adolescence-limited delinquency lie in youngsters’ best efforts to cope with the widening gap between biological and social maturity.' (Moffitt, 1993, p. 692). Moffitt invoked the concept of a maturity gap to explain the behaviour of the AL group – essentially, they wish to be treated like adults, but society treats them as children, so they imitate their LCP antisocial peers in a misguided attempt to obtain status and the privileges of adulthood (e.g., access to alcohol). Consequently, these groups are considered to differ qualitatively, rather than quantitatively – LCP antisocial behaviour is a form of psychopathology, whereas AL antisocial behaviour is viewed as virtually normative. In line with this view, Moffitt predicted that LCP antisocial behaviour would be relatively rare, whereas AL antisocial behaviour would be common amongst adolescent populations.

In the two decades since its publication, the developmental taxonomic theory has had a major influence on the fields of developmental psychology, psychiatry, education and criminology. At the time of writing this review, the 1993 article describing the theory has been cited over 5,000 times according to Google Scholar, making it one of the most highly cited papers in psychology. It has inspired several fruitful lines of research and helped to bring together the disciplines of developmental psychology, psychopathology and criminology. In addition, the distinction set out in the theory between LCP and AL antisocial behaviour has informed the categories of childhood-onset and adolescent-onset conduct disorder that were first incorporated into the DSM-IV in 1994 (American Psychiatric Association, 1994), and have been retained in the recent DSM-5 (American Psychiatric Association, 2013). The purpose of this review was to provide a timely overview and critique of the developmental taxonomic theory, in the light of recent empirical studies which indicate that revision and reformulation of some aspects of the theory may be required.

In addition to its influence on a variety of disciplines, the developmental taxonomic theory has wide-ranging implications for research on antisocial behaviour and clinical practice with children with disruptive behaviour disorders. First of all, as the theory has informed the distinction between childhood-onset and adolescent-onset forms of conduct disorder set out in the DSM-IV and DSM-5, the validity of the theory is important for our diagnostic and classification systems. Specifically, is age-of-onset a meaningful way to parse the heterogeneity within antisocial behaviour, and is it useful for clinicians to make this distinction when assessing patients and formulating treatment strategies? Second, if there are *qualitative* differences in aetiology between LCP and AL forms of antisocial behaviour, this must be taken into account by researchers studying antisocial behaviour. In particular, the theory implies that researchers must clearly distinguish between LCP and AL antisocial behaviour when studying groups of adolescents. If they do not do so, their studies may dramatically underestimate the contribution of neurobiological or genetic influences on LCP antisocial behaviour. Consequently, the theory suggests that neurobiological research should focus on the LCP group, because including individuals with AL antisocial behaviour may obscure group differences and unnecessarily increase heterogeneity of the antisocial group. Third, in terms of clinical practice, the view that LCP antisocial behaviour is a form of psychopathology whereas AL antisocial behaviour is virtually normative implies that clinicians should devote their energies to treating or developing new interventions for the LCP group. Conversely, the theory can be interpreted as implying that individuals with AL antisocial behaviour are more likely to respond to psychological therapies, whereas LCP individuals will be less responsive to treatment as the cumulative consequences of their antisocial behaviour have been building up over many years.

In the remainder of this review, we will discuss epidemiological, psychometric, neuropsychological, neuroendocrinological, and neuroimaging research testing the developmental taxonomic theory, and evaluate the extent to which the findings of these diverse studies support the theory. It should be noted that there will be some variation regarding the terms used to describe patterns of antisocial behaviour: ‘LCP’ and ‘AL’ are appropriate when considering participants in longitudinal studies who have been followed into adulthood, whereas cross-sectional studies cannot state with certainty that childhood-onset patterns of antisocial behaviour will be life-course persistent, nor can they tell whether an adolescence-onset pattern will be adolescence-limited, if the participants are tested during adolescence. Consequently, we will use the terms LCP and AL antisocial behaviour when considering findings from prospective longitudinal studies, but our default position, especially when describing cross-sectional studies (which make up the majority of the studies included in this review) will be to use the terms ‘childhood-onset’ and ‘adolescence-onset’ CD. While these terms are not synonymous, there is considerable overlap between LCP and childhood-onset antisocial behaviour, and also between AL and adolescence-onset antisocial behaviour.

## Methods

Relevant studies were identified using PubMed and Web of Science to perform literature searches with the keywords ‘developmental taxonomic theory’, ‘developmental taxonomy’, ‘childhood-onset conduct disorder’, ‘early-onset conduct disorder’ and ‘adolescent-onset conduct disorder’. We restricted our search to studies published in English, with a search period covering 1993–2013. We also searched in the reference sections of key review articles and papers from the Dunedin Multidisciplinary Health and Development Study. Where possible, we focused on empirical studies which distinguished between childhood-onset and adolescence-onset subtypes of CD or between life-course persistent and adolescence-limited forms of antisocial behaviour. This literature search yielded 61 empirical studies directly relevant to the developmental taxonomic theory in the areas of epidemiology, personality assessment, genetics, neuropsychology, neuroendocrinology and neuroimaging (these studies are denoted by an asterisk in the reference list).

## Epidemiological evidence relevant to the developmental taxonomic theory

The primary source of the findings that led to the theory was a longitudinal birth cohort investigation, the Dunedin Multidisciplinary Health and Development Study. Using age 18 follow-up data from this study, Moffitt and colleagues were able to identify the two hypothesised groups of adolescent offenders described in the theory: one whose behavioural difficulties started in early childhood, and another whose antisocial behaviour emerged during adolescence (Moffitt, Caspi, Dickson, Silva, & Stanton, 1996). Childhood predictors of negative outcomes such as hyperactivity, low verbal IQ or low socioeconomic status were found to be far more common in the LCP than the AL group, even though these groups showed similar rates of criminal offending at age 15 (Moffitt & Caspi, 2001; Moffitt, Caspi, Rutter, & Silva, 2001). However, Moffitt et al. (1996) also identified a ‘recovery’ group of equal size to the LCP group, and subsequent studies have confirmed the existence of a third trajectory of antisocial behaviour: the childhood-limited (CL) pathway, in which individuals exhibit severe conduct problems in early childhood, but desist from engaging in antisocial behaviour in adolescence (Aguilar, Sroufe, Egeland, & Carlson, 2000; Fergusson, Horwood, & Nagin, 2000; Odgers et al., 2007, 2008; Raine et al., 2005). Interestingly, many population-based studies have shown that childhood-limited antisocial behaviour is actually the most common outcome of childhood-onset conduct problems (Barker & Maughan, 2009; Nagin & Tremblay, 1999; Odgers et al., 2007, 2008). Thus, whilst the terms childhood-onset and LCP antisocial behaviour were used interchangeably in Moffitt's early writings, subsequent research has demonstrated that 50%–70% of individuals with childhood-onset conduct problems outgrow their difficulties by adolescence.

In addition to studies documenting the existence of a childhood-limited antisocial group, recent work has reported an association between childhood adversity and the development of antisocial behaviour in adolescence (Fergusson et al., 2000; Roisman, Monahan, Campbell, Steinberg, & Cauffman, 2010). For example, a recent prospective longitudinal study that assessed antisocial behaviour repeatedly throughout childhood and adolescence found little evidence for differences between childhood-onset persistent (similar to LCP), adolescence-onset and childhood-limited antisocial groups in exposure to childhood adversity or intraindividual risk factors (Roisman et al., 2010). Interestingly, however, all three groups were elevated on these risk variables relative to controls. This result is consistent with the findings of Fergusson et al. (2000), who showed that exposure to psychosocial adversity was higher for all groups on an antisocial trajectory (including the adolescence-onset group) compared with controls, although childhood-onset offenders were exposed to the highest levels of adversity and maladaptive family functioning (Fergusson et al., 2000).

Further challenges to the developmental taxonomic theory have come from studies investigating adult outcomes of adolescence-onset antisocial behaviour which, according to the theory, should typically follow an ‘adolescence-limited’ course. In an important study by Odgers et al. (2007), using data from the Dunedin Study, there were no differences in conduct problems between the adolescence-onset and LCP groups at age 26 (the last wave of the study when the level of conduct problems were measured). More importantly, the age 32 follow-up data showed that mental and physical health outcomes were highly negative for both the LCP and adolescence-onset groups, relative to controls. The adolescence-onset group was impaired relative to the control group on 9/15 mental health and 7/14 physical health outcome measures (in comparison, the LCP group was impaired on 13/15 mental health and 12/14 physical health measures). Adolescence-onset individuals were also more likely than controls to report engaging in violence or partner abuse, or to have an adult conviction for violence. These findings contradict the notion that antisocial behaviour beginning in adolescence will remit as the individual enters adulthood, and suggest that the ‘adolescence-limited’ term is a misnomer.

Recent research has investigated whether the behaviours that make up the DSM-IV CD diagnosis show similar or different developmental trajectories and relationships with adult criminality. A prospective longitudinal study which followed a cohort of males from ages 12 to 31 found that aggression and theft (i.e., a form of rule-breaking delinquency) showed distinct developmental trajectories (Barker et al., 2007). Theft increased in frequency over adolescence in the majority of the cohort (55%), whereas only a small subgroup (13%) showed increases in aggressive behaviour over the same period. Several other studies found that aggression decreases in frequency with age in most individuals, whereas theft becomes more common during the teenage years (Nagin & Tremblay, 1999), suggesting that these subtypes of antisocial behaviour may have distinct aetiologies and should be studied separately (Tremblay, 2010). To further investigate these issues, a recent study by Burt, Donnellan, Iacono and McGue (2011) examined whether age-of-onset of CD or the subtypes of antisocial behaviour shown by the individual (aggression or rule-breaking) was a better predictor of adult antisocial outcomes. Consistent with the developmental taxonomic theory, they found that individuals with childhood-onset CD that persisted into adolescence were more likely to meet criteria for antisocial personality disorder (APD) in adulthood than individuals who developed CD during adolescence. However, 15.5% of the latter group still fulfilled criteria for APD at age 24, as compared with 54.2% of the former. Interestingly, when the authors controlled for the behavioural subtypes manifested by the individual, CD age-of-onset no longer predicted adult APD. These results suggest that the forms of antisocial behaviour that the person displays (aggression vs. rule-breaking) are more important in terms of predicting persistence into adulthood than age-of-onset of CD. In addition, rule-breaking in adolescence was the strongest individual predictor of adult APD symptoms, rather than aggression.

Another important study applied latent class analysis to a very large epidemiological dataset to explore the heterogeneity of CD: it identified *five* distinct classes of CD (Nock, Kazdin, Hiripi, & Kessler, 2006). Interestingly, only the ‘pure aggressive’ class of CD had a mean age of onset below age 10; the most severe and impairing classes of CD (termed ‘severe covert’ and ‘pervasive’) both had mean ages of onset above 11 years. This suggests that age-of-onset of CD is not related to severity of antisocial behaviour in the manner predicted by the developmental taxonomic theory or the DSM-IV criteria. The study also showed that the most severe classes of CD were associated with the greatest risk for subsequent mental disorders. One limitation of this study was its reliance on retrospective reports of CD symptoms and their age-of-onset.

In addition, epidemiological data have raised the question of whether the developmental taxonomic theory applies equally to males and females. Silverthorn and Frick (1999) argued that, as girls only rarely show childhood-onset antisocial behaviour but come close to catching up with their male peers by midadolescence (Cohen, Cohen, & Brook, 1993; Lahey et al., 2000; Moffitt et al. 2001), females may follow a third developmental pathway to antisocial behaviour: the delayed-onset pathway. According to this model, the personality and neuropsychological characteristics which distinguish male childhood-onset individuals, such as psychopathic traits, are also observed in females with adolescence-onset antisocial behaviour. This model has received some empirical support from studies documenting similar personality traits and impulse control difficulties in childhood-onset CD males and adolescence-onset CD females (Silverthorn, Frick, & Reynolds, 2001). Subsequent prospective longitudinal studies have shown that childhood-onset CD or the LCP trajectory does exist in females, but it is relatively rare (with sex ratios between 3:1 and 15:1 in favour of males; Kratzer & Hodgins, 1999; Moffitt et al., 2001; Lahey et al., 2006; Odgers et al., 2008; although for conflicting results from a high-risk sample see Keenan, Wroblewski, Hipwell, Loeber, & Stouthamer-Loeber, 2010). These studies have also revealed that females may show either an adolescence-delayed-onset or an adolescence-limited trajectory (Fontaine, Carbonneau, Vitaro, Barker, & Tremblay, 2009), and that adolescence-onset females are at increased risk for negative adult outcomes relative to their peers even though they are likely to desist from showing antisocial behaviour in adulthood (Odgers et al., 2008). Intriguingly, Odgers et al. (2008) found that females largely appeared to follow an adolescence-limited trajectory, whereas adolescence-onset conduct problems were far more likely to persist into adulthood in males. This suggests that the LCP versus AL distinction may actually apply better to females than males.

The final study we will consider in this section was performed by Walters (2011), who applied taxometric analyses to data on the externalising symptoms displayed by a large sample of individuals with antisocial behaviour, to examine whether the LCP and AL groups could be distinguished empirically. There was no evidence for a taxonic (i.e., categorical) boundary between LCP and AL forms of antisocial behaviour, suggesting that the differences between these subtypes are quantitative, rather than qualitative, in nature. This challenges the developmental taxonomic theory which holds that these subtypes are aetiologically distinct, instead indicating that they both fall on the same underlying dimension, with LCP individuals merely higher on the antisocial dimension than AL individuals (Walters, 2011).

In summary, recent epidemiological data have challenged the developmental taxonomic theory in several ways: (a) there is a third trajectory of antisocial behaviour, termed the ‘childhood-limited pathway’, which is marked by childhood-onset conduct problems, but desistance from serious antisocial behaviour by adolescence; (b) the term ‘adolescence-limited’ antisocial behaviour appears to be a misnomer, as many individuals with adolescence-onset CD continue to show severe antisocial behaviour and experience poor mental and physical health outcomes as adults; (c) the developmental antecedents of adolescence-onset/AL antisocial behaviour appear to differ only quantitatively, rather than qualitatively, from those of LCP antisocial behaviour, as both groups experience higher levels of psychosocial adversity than controls; (d) aggressive and rule-breaking forms of antisocial behaviour show different developmental trajectories, with rule-breaking more strongly associated with adult APD than the age-of-onset of CD; (e) it is unclear whether the developmental taxonomic theory applies equally well to females and males, as the course of CD appears to differ by sex, with females rarely showing LCP or childhood-onset CD, leading some researchers to propose the existence of a ‘delayed-onset’ antisocial pathway in females; and (f) taxometric analyses suggest that differences between LCP and AL antisocial behaviour are quantitative, rather than qualitative, in nature.

## Personality trait correlates of CD

Moffitt et al. (1996) measured personality profiles at age 18 in the Dunedin cohort, using the Multidimensional Personality Questionnaire (Tellegen, 1982), finding that the LCP group scored lower on personality traits related to affiliative behaviour, and higher in aggression, impulsivity, and stress reaction than controls. The AL group also differed from controls on several of these measures. The only variable that differed between the LCP and AL groups was social closeness, a personality trait reflecting sociability and a desire to affiliate with others, which was lower in LCP individuals. In a subsequent follow-up at age 26, Moffitt, Caspi, Harrington, and Milne (2002) found that AL individuals scored higher in negative emotionality, and lower in agreeableness, constraint, and conscientiousness than controls. Although the LCP group scored higher than the AL group on negative emotionality, and lower on agreeableness and social closeness, the AL group scored lower than the LCP group on constraint (reflecting increased impulsivity). These findings suggest that AL individuals show a distinct constellation of personality traits that, while less pathological than the LCP group, nevertheless places them at elevated risk for psychopathology and interpersonal problems. Consistent with the latter results, several other studies reported that adolescence-onset and childhood-onset antisocial behaviour are both associated with increased impulsivity relative to controls, or failed to detect differences in impulsivity between these groups (Aguilar et al., 2000; Dandreaux & Frick, 2009; Taylor, Iacono, & McGue, 2000; White, Bates, & Buyske, 2001).

A number of studies have investigated psychopathic or callous-unemotional traits in adolescents with CD. In three separate male samples, we found that both childhood-onset and adolescence-onset forms of CD were associated with elevated psychopathic and callous-unemotional traits relative to healthy controls, whereas there were no significant differences between these CD subgroups on either measure (Fairchild, van Goozen, Calder, Stollery, & Goodyer, 2009; Fairchild, van Goozen, Stollery, Brown et al., 2008; Fairchild et al., 2011). We have also shown in two separate studies that females with adolescence-onset CD show elevated psychopathic and callous-unemotional traits relative to controls (Fairchild, Stobbe, van Goozen, Calder, & Goodyer, 2010; Fairchild et al., 2013). In contrast to these findings, however, Dandreaux and Frick (2009) found that childhood-onset offenders had higher levels of callous-unemotional traits than adolescence-onset offenders, although unfortunately no control group was included in this study (Dandreaux & Frick, 2009).

In summary, personality research has provided little support for the contention that there are qualitative differences in personality traits between AL/adolescence-onset and LCP groups, or the proposal that only LCP individuals will exhibit atypical personality profiles. Most studies in this area have shown that AL or adolescence-onset CD individuals differ from controls on multiple personality variables such as impulsivity and negative emotionality, contradicting the developmental taxonomic theory, although personality profiles are generally more extreme in LCP or childhood-onset groups. In addition, while there are exceptions to this pattern, several studies have reported elevated levels of psychopathic and callous-unemotional traits in both childhood-onset and adolescence-onset forms of CD.

## Behavioural and molecular genetics of CD

Very few behavioural genetic studies have been designed to test the developmental taxonomic theory by directly comparing the heritability of LCP and AL forms of antisocial behaviour using prospective longitudinal data. However, several studies have investigated whether the relative contribution of genetic and environmental influences on antisocial behaviour changes across the life span. The developmental taxonomic theory can be interpreted as predicting that genetic influences should follow an U-shaped function with age: these should be strongest in childhood and adulthood, reflecting the fact that LCP is under genetic influence, and weakest during adolescence, as the latter measure would be confounded by the presence of a high proportion of AL individuals whose antisocial behaviour arises from environmental, rather than genetic, origins.

Rhee and Waldman (2002) performed a meta-analysis of behavioural genetic studies of antisocial behaviour, but were unable to investigate whether LCP antisocial behaviour was more heritable than adolescence-onset/AL forms of antisocial behaviour, as insufficient longitudinal data were available to enable them to test this hypothesis. However, their meta-analysis found that the heritability of antisocial behaviour was greater in childhood than in adolescence or adulthood, contrary to the U-shaped function predicted by the developmental taxonomic theory. In contrast, an earlier meta-analysis found that genetic influences on antisocial behaviour increased and shared environmental influences decreased between childhood and adulthood (Miles & Carey, 1997), again diverging from the U-shaped pattern predicted by the theory. We will now consider specific behavioural genetic studies which are particularly relevant to the developmental taxonomic theory.

In an important twin study, Taylor et al. (2000) explicitly compared the heritability of childhood-onset and adolescence-onset forms of CD, finding evidence for genetic influences on the former, but not the latter, subtype of CD. The authors also found higher rates of adult antisocial behaviour in the first- and second-degree relatives of the childhood-onset CD participants than the relatives of adolescence-onset CD or control participants. Interestingly, however, 95% of the cotwins who were concordant for CD showed the same subtype of CD, indicating that when genetically-identical individuals are concordant for CD they are likely to develop the same variant. Although limited by a relatively small sample size (*n* = 70 twin pairs with at least one cotwin meeting DSM-III-R criteria for CD), this study provides strong support for the developmental taxonomic theory.

In a subsequent study of 1,186 twin pairs, Eley, Lichtenstein, and Moffitt (2003) found that aggressive antisocial behaviour in childhood was highly heritable (0.60), whereas aggressive antisocial behaviour in adolescence was only moderately heritable (0.46). Non-aggressive antisocial behaviour was moderately heritable when considering either childhood or adolescence (0.49 and 0.44, respectively). Shared environmental influences on nonaggressive antisocial behaviour were moderate in size at both time points (0.35 and 0.42 for childhood and adolescence, respectively). Overall, this study suggested that childhood antisocial behaviour was more heritable than adolescent antisocial behaviour; this was particularly true for aggression. These results provide some support for the developmental taxonomic theory, but they do not appear to show a qualitative difference between childhood and adolescence in the heritability of aggression and antisocial behaviour.

In a direct test of the developmental taxonomic theory, Silberg, Rutter, Tracy, Maes, and Eaves (2007) investigated genetic and environmental influences on antisocial behaviour at several points during adolescence in a sample of 1,037 male twins. They found that heritability estimates were highest (0.43) for antisocial behaviour in early adolescence and lowest (0.05) in midadolescence. There was also a transient genetic effect on antisocial behaviour displayed around the pubertal transition. In contrast, shared environmental influences were largest in midadolescence, demonstrating the importance of environmental factors in the aetiology of adolescence-onset antisocial behaviour. These results are in line with the developmental taxonomic theory.

Jacobson, Prescott, and Kendler (2002) measured genetic and environmental influences on antisocial behaviour in a large sample of male and female twins (*n* = 6,806). They found that genetic influences on antisocial behaviour increased from childhood (defined as below age 15) to adolescence and adulthood. Notably, for male twins, genetic influences on childhood antisocial behaviour were weak (0.06), whereas genetic influences on antisocial behaviour in adolescence or adulthood were moderate in size (0.41 and 0.40, respectively). The study also observed unique genetic influences on adolescent antisocial behaviour that were not shared with childhood antisocial behaviour, possibly reflecting genetic influences on biological processes activated during puberty. These results are not consistent with the developmental taxonomic theory.

In another recent study, Van Hulle et al. (2009) found that genetic influences on antisocial behaviour in adolescence were distinct from those affecting antisocial behaviour in childhood, although heritable influences were significant at both time points. The authors interpreted their findings as evidence that youth showing persistent antisocial behaviour from childhood into adolescence are influenced by one set of genetic factors, whereas a second set of genetic factors affect antisocial behaviour which emerges around the pubertal transition. These observations run counter to the developmental taxonomic theory, as they suggest that adolescence-onset CD may have a genetic aetiology (albeit one that is distinct from childhood-onset CD).

Taking a different perspective, Burt and Neiderhiser (2009) examined whether genetic influences on rule-breaking and aggressive antisocial behaviours declined or increased over the adolescent period. Their findings demonstrated that heritability of rule-breaking forms of delinquency increased in the transition from early- to late-adolescence, whereas genetic influences on aggression remained stable over this period. Interestingly, shared environmental influences were most pronounced in childhood and declined dramatically in midadolescence. In addition to suggesting differential genetic influences on different forms of antisocial behaviour, these results demonstrated distinct heritable influences on adolescence-onset antisocial behaviours involving rule-breaking.

Burt (2009) subsequently performed a meta-analysis investigating genetic and environmental influences on aggressive and rule-breaking antisocial behaviour. She found that aggressive behaviour was more heritable than rule-breaking behaviour (0.65 vs. 0.48, respectively). Rule-breaking behaviour was influenced by the shared environment (0.16), whereas shared environmental influences on aggression were weak. These results suggest aetiological differences between aggressive and nonaggressive forms of antisocial behaviour, consistent with the studies described above demonstrating that these behavioural subtypes show distinct developmental trajectories. The results also have implications for the developmental taxonomic theory, as aggression is more strongly associated with LCP than adolescence-onset/AL antisocial behaviour.

In conclusion, behavioural genetic studies have yielded mixed results with some studies showing that the heritability of antisocial behaviour increases, whereas shared environmental influences decrease, with age. On the other hand, several studies have reported that the heritability of antisocial behaviour is highest in childhood and lowest in adulthood. Neither pattern is consistent with the developmental taxonomic theory, which predicts that genetic influences on antisocial behaviour will be strongest in childhood and adulthood, and weakest in adolescence (as only LCP antisocial behaviour is considered heritable). While several twin studies have provided support for an aetiological distinction between childhood-onset and adolescence-onset antisocial behaviour, recent studies have revealed unique genetic influences on antisocial behaviours that emerge during adolescence (particularly rule-breaking delinquency) or puberty. Consequently, behavioural genetic studies have provided only equivocal support for the developmental taxonomic theory.

Molecular genetic studies have documented a robust and replicable interaction between the monoamine oxidase-A (*MAOA*) gene and childhood maltreatment in the aetiology of antisocial behaviour (Caspi et al., 2002; Fergusson, Boden, Horwood, Miller, & Kennedy, 2012; Kim-Cohen et al., 2006). However, the majority of these studies appear to have collapsed across childhood-onset and adolescence-onset forms of CD or studied children only. Therefore, to our knowledge, no studies have examined whether this interaction between maltreatment and *MAOA* genotype is specific to childhood-onset CD, as would be predicted by the developmental taxonomic theory.

## Cortisol secretion and stress reactivity in CD

The relationship between antisocial behaviour and hypothalamic-pituitary-adrenal (HPA) axis activity is complex and findings in the literature have been mixed (Alink et al., 2008; van Goozen, Fairchild, Snoek, & Harold, 2007). However, studies focusing on school-aged children have largely observed a negative relationship between basal or stress-induced cortisol and antisocial behaviour, which appears to be strongest in clinic-referred samples.

McBurnett, Lahey, Rathouz, and Loeber (2000) studied the relationship between CD, aggression, and basal cortisol levels in a sample of clinic-referred boys. They found that boys with childhood-onset CD had lower basal cortisol levels than those with adolescence-onset CD, which appeared to be explained by higher levels of aggression in the former group. Unfortunately, this study did not control for time of day of saliva collection, which makes it difficult to interpret these results because cortisol secretion shows a pronounced circadian rhythm. A recent study reported that life-course persistent, childhood-limited, and adolescence-onset forms of conduct disorder were all associated with lower waking cortisol levels, whereas none of these groups differed from each other (Haltigan, Roisman, Susman, Barnett-Walker & Monahan, 2011). This study is important because it employed a prospective longitudinal design with measurements of antisocial behaviour obtained from age 5 onwards, and time of saliva sampling was standardised across participants. In our research investigating basal cortisol secretion, we found no differences between childhood-onset or adolescence-onset CD participants and controls in morning cortisol levels, although both CD groups showed elevated evening cortisol levels relative to controls, suggesting a flatter circadian profile in CD (Fairchild, van Goozen, Stollery, Brown et al., 2008).

In relation to cortisol reactivity, early studies showed that children with disruptive behaviour disorders showed blunted cortisol responses to stress (van Goozen, Matthys, Cohen-Kettenis, Buitelaar, & van Engeland, 2000; van Goozen et al., 1998), particularly if they were also low in anxiety. These findings suggest that childhood-onset CD is associated with reduced HPA axis responses to stress. We recently extended these findings by demonstrating that both childhood-onset and adolescence-onset forms of conduct disorder were associated with reduced cortisol responses to a psychological stressor involving provocation and frustration (Fairchild, van Goozen, Stollery, Brown et al., 2008). There were no significant differences in cortisol reactivity between these subgroups. Overall, these studies suggest that basal cortisol secretion and cortisol reactivity to stress are altered in both childhood-onset and adolescence-onset forms of CD, which is inconsistent with the developmental taxonomic theory.

## Neuropsychological and psychophysiological studies of CD

Moffitt (1993) originally focused on verbal and executive functions when specifying the neuropsychological impairments involved in the aetiology of LCP. However, she also noted that neuropsychological variation might underlie individual differences between children in temperament, emotional reactivity, impulse control and cognitive abilities, leading to a heightened risk for antisocial behaviour. In subsequent years, a consensus has emerged that severe antisocial behaviour is associated with impairments in emotion recognition and regulation. In contrast, the evidence for deficits in executive function in antisocial behaviour has been more mixed, especially when studies have controlled for intelligence quotient (IQ) differences between groups.

### Intelligence

One of the most robust findings in criminology is that delinquents or children with severe antisocial behaviour have lower IQ scores than nondelinquents (Rutter, Giller, & Hagell, 1998). Early work investigating IQ in delinquents found that the 8-point difference in full-scale IQ between delinquents and nondelinquents masked a striking difference between subtypes: stable, childhood-onset delinquents had a 17-point reduction in mean IQ, whereas ‘temporary’ (adolescence-limited) delinquents showed only a 1-point reduction (Moffitt, 1990).

Using data from the Pittsburgh Youth Study, Raine et al. (2005) found that out of 15 different measures of neuropsychological function spanning verbal, spatial and executive functions, the LCP group differed significantly from controls on 7/15 measures, whereas the AL group differed from controls on 3/15 measures. However, when directly comparing these groups, the AL group differed significantly from the LCP group on just a single measure: verbal IQ. While consistent with the proposal that neuropsychological impairments should be most marked in those with LCP antisocial behaviour, these data do not support the contention that LCP and AL forms of antisocial behaviour are qualitatively different from a neuropsychological perspective.

Kratzer and Hodgins (1999) studied a Swedish birth cohort, relating different aspects of IQ, as measured at age 13, to developmental trajectories of antisocial behaviour. Although LCP males scored lower on all of the IQ subtests than AL offenders, adult starter offenders, and controls, the AL group were also impaired on all IQ measures relative to controls. These prospective data again suggest that IQ differences between LCP and AL offenders are quantitative rather than qualitative, and that both forms of antisocial behaviour are associated with IQ deficits.

### Executive functions and decision-making

In an important prospective longitudinal study, Aguilar et al. (2000) investigated the neurodevelopmental and social origins of LCP and adolescence-onset antisocial behaviour. They found that the LCP and adolescence-onset groups differed in rates of exposure to adversity, but not in temperament or neuropsychological functioning, in early childhood. Differences between these groups in neuropsychological functioning were only observed in late childhood or adolescence, which was interpreted as evidence that neuropsychological deficits in LCP individuals were secondary to exposure to childhood adversity (Aguilar et al., 2000).

Using cross-sectional designs, Pajer et al. (2008) and Giancola, Mezzich, and Tarter (1998) both investigated neuropsychological function in adolescent girls with CD, finding impairments in multiple aspects of executive function and visuospatial processing and lower IQ in the CD groups relative to controls. However, neither study observed a relationship between CD age-of-onset and severity of neuropsychological deficits, contrary to the developmental taxonomic theory.

van Goozen, Cohen-Kettenis et al. (2004) found no evidence for neuropsychological deficits in executive function or inhibitory control in children with oppositional defiant disorder (ODD) or CD; rather, the group differences they observed were specific to a task with a motivational inhibitory component. Participants with childhood-onset ODD or CD were more likely to carry on playing despite receiving an increasing ratio of losses to gains as the task progressed, suggesting that they were less sensitive to punishment.

In a study examining decision-making under risk in CD, we found that male adolescents with both childhood-onset and adolescence-onset forms of CD made more risky decisions than controls (Fairchild, van Goozen, Stollery et al., 2009). Interestingly, the participants with CD systematically adjusted their behaviour in light of the rewards and losses available in each trial, but were more likely than controls to select the risky choice across a range of choices varying in terms of the probability and the size of potential gains and losses. These results imply that individuals with both forms of CD are either less sensitive to potential losses or more sensitive to gains, or both. In the same study, neither CD subgroup showed a deficit in ‘cold’ executive function (as measured using the Wisconsin Card Sorting Test) when factoring out group differences in IQ. Considered together, these studies provide little support for differences in executive function between childhood-onset and adolescence-onset forms of CD. Rather, these studies suggest that both CD subgroups show altered decision-making under ‘hot’ or motivational conditions.

### Emotion recognition, emotional reactivity and fear conditioning

As mentioned above, there is increasing agreement that deficits in emotion recognition and emotional reactivity/regulation are associated with antisocial behaviour (Davidson, Putnam & Larson, 2000). Consistent with this view, we found that males with childhood-onset CD and females with adolescence-onset CD showed impaired recognition of angry and disgusted facial expressions (Fairchild, van Goozen, Calder et al., 2009; Fairchild et al., 2010). In the study focusing on males, adolescence-onset CD was associated with similar impairments to those observed in childhood-onset CD, but most comparisons between adolescence-onset CD individuals and controls did not reach a corrected level of significance. Notably, there were no significant differences between the childhood-onset and adolescence-onset CD groups in facial emotion recognition.

Psychophysiological studies have reported reduced electrodermal responses to affective and neutral images (Herpertz et al., 2005) and reduced startle responses to an acoustic probe in children or adolescents with childhood-onset CD (Fairchild, van Goozen, Stollery, & Goodyer, 2008; van Goozen, Snoek, Matthys, van Rossum, & van Engeland, 2004). These findings appear to reflect a generalised deficit in autonomic reactivity, rather than a valence-specific effect (i.e., reduced responses to negatively valenced stimuli). When investigating affective modulation of the startle reflex in adolescence-onset CD, we found that this group also showed reduced startle responses when viewing all slide types compared with controls (Fairchild, van Goozen, Stollery, & Goodyer, 2008). Moreover, these reductions were numerically greater than those observed in the childhood-onset CD group. In a subsequent study, we found that girls with adolescence-onset CD also showed reduced startle responses relative to controls, again irrespective of slide valence (Fairchild et al., 2010). We also investigated autonomic fear conditioning in childhood-onset and adolescence-onset forms of CD, finding statistically equivalent reductions in fear conditioning in both groups in males (Fairchild, van Goozen, Stollery, & Goodyer, 2008). In addition, females with adolescence-onset CD showed impaired autonomic conditioning relative to controls (Fairchild et al., 2010). Lastly, heart rate responses to psychosocial stress were attenuated in males with both childhood-onset and adolescence-onset forms of CD (Fairchild, van Goozen, Stollery, Brown et al., 2008). Considered together, these findings suggest that both childhood-onset and adolescence-onset variants of CD are associated with impairments in emotion recognition, fear conditioning, and autonomic reactivity.

In conclusion, there is now considerable evidence for neuropsychological impairments in LCP antisocial behaviour or childhood-onset CD. These findings go beyond early predictions of deficits in IQ or executive function in LCP antisocial behaviour to document impairments in facial emotion recognition, decision-making and emotional reactivity in CD. However, in many cases, similar neurocognitive and psychophysiological impairments have been reported in adolescence-onset CD. In some studies, the adolescence-onset CD group actually showed greater impairments than the childhood-onset CD group, although in most studies they performed at an intermediate level between controls and childhood-onset CD participants. Consequently, this work supports the view that adolescence-onset CD is quantitatively, rather than qualitatively, different from childhood-onset CD.

## Structural and functional neuroimaging studies of CD

Progress in understanding the neurobiological basis of antisocial behaviour has accelerated in recent years, with a substantial number of studies using structural or functional neuroimaging techniques such as magnetic resonance imaging (MRI) to investigate CD. The majority of these studies have been restricted to individuals with childhood-onset CD, probably due to the influence of the developmental taxonomic theory.

### Structural imaging studies

In one of the first studies in this area, Kruesi, Casanova, Mannheim and Johnson-Bilder (2004) investigated grey and white matter volume in male adolescents with childhood-onset CD. The authors found reduced grey matter in the temporal lobe of the CD group relative to healthy controls. However, the groups used were relatively small (*n* = 10) and differed in IQ. The analyses also relied on manual tracing of brain structures and were unable to localise the changes to specific regions of the temporal lobe such as the amygdala. Sterzer, Stadler, Poustka and Kleinschmidt (2007) used voxel-based morphometry, an unbiased, automated method which provides more detailed information about regional differences, to study grey matter volume in male adolescents with childhood-onset CD and comorbid ADHD. They found that the childhood-onset CD group showed reduced amygdala and anterior insula volume compared with controls. Huebner et al. (2008) found that male adolescents with childhood-onset CD (most of whom had comorbid ADHD) showed volumetric reductions in left orbitofrontal cortex and left amygdala extending to surrounding medial temporal lobe regions, such as the hippocampus. A recent structural imaging study observed reduced anterior insula and medial prefrontal cortex volume in eight year-old children with ODD or CD relative to controls (Fahim et al., 2011).

**Figure 2:**
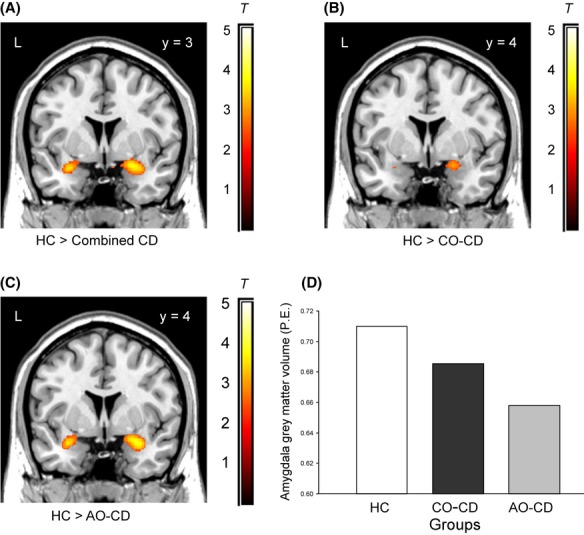
Group differences in amygdala grey matter volume: (A) grey matter volume was reduced in bilateral amygdala in a combined group of adolescents with conduct disorder (*n* = 63), relative to healthy controls (HCs; *n* = 27); (B) significant bilateral amygdala volume reduction in participants with childhood-onset conduct disorder (CO-CD) relative to HCs; and (C) significant bilateral amygdala volume reduction in participants with adolescence-onset conduct disorder (AO-CD) relative to HCs. The colour bars, ranging from red to white, represent T statistics. Panel D depicts mean values for right amygdala grey matter volume in each group. (From Fairchild et al., 2011, *American Journal of Psychiatry*, 168(6), p. 628; Copyright American Psychiatric Association, 2011.)

Our recent structural MRI study replicated the above findings of reduced amygdala volume in males with childhood-onset CD (Fairchild et al., 2011). Crucially, however, we also observed bilateral reductions in amygdala volume in males with adolescence-onset CD (see Figure 2). These results remained significant when controlling for ADHD symptoms, suggesting that reductions in amygdala volume were not explained by ADHD comorbidity. This latter observation is consistent with the results of a voxel-based morphometry study which directly compared adolescents with noncomorbid CD and noncomorbid ADHD, finding widespread structural abnormalities in the CD group relative to both the control and ADHD groups (Stevens & Haney-Caron, 2012). In a further study, we demonstrated that females with adolescence-onset CD also showed reduced grey matter volume in the anterior insula compared with healthy controls (Fairchild et al., 2013). Finally, a recent study observed reduced cortical thickness in widespread temporal and parietal regions, and reduced folding in insula and prefrontal cortex in both childhood-onset and adolescence-onset forms of CD (Hyatt, Haney-Caron, & Stevens, 2012). Interestingly, when directly comparing these subtypes, adolescence-onset CD participants showed reduced cortical folding in the insula and ventromedial prefrontal cortex relative to childhood-onset CD participants. Considered together, these results indicate that both forms of CD are associated with reductions in grey matter volume and cortical thickness in brain regions implicated in emotion processing and regulation, such as the amygdala, anterior insula, and orbitofrontal cortex. These structural changes may underlie the neuropsychological deficits observed in both subtypes of CD, as described in the section above.

Recent studies have used diffusion tensor imaging methods to investigate anatomical connectivity in the uncinate fasciculus white-matter pathway that connects the prefrontal cortex and the amygdala (and surrounding anterior temporal lobe) in adolescents with CD. Surprisingly, these initial studies have found that the microstructural integrity of the uncinate fasciculus is increased in adolescents with CD (Passamonti et al., 2012; Sarkar et al., 2013; although see Finger et al., 2012). These changes may have functional consequences, as the uncinate fasciculus is implicated in emotion regulation and shows reduced microstructural integrity in individuals with affective disorders (Tromp do et al., 2012). The study by Passamonti et al. (2012) focused specifically on males with childhood-onset CD, whereas Sarkar et al. (2013) included both sexes and did not differentiate between childhood-onset and adolescence-onset subtypes of CD. It is interesting to note that these results are in the opposite direction to those obtained in adults with psychopathy or antisocial personality disorder, who show reduced microstructural integrity in the uncinate fasciculus (Craig et al., 2009; Sundram et al., 2012). As the typical pattern of white-matter development in the uncinate fasciculus is an inverted-U function with age (Lebel et al., 2012), one possible explanation of these findings is that individuals with CD show accelerated maturation of the uncinate fasciculus tract in childhood or adolescence, which is followed by earlier or more pronounced reductions in microstructural integrity in adulthood (Passamonti et al., 2012). These anatomical connectivity results are consistent with the view that childhood-onset CD is a neurodevelopmental disorder, although further studies are needed to confirm these preliminary findings in larger samples and assess the microstructural integrity of white-matter pathways in adolescence-onset CD. On the basis of prior volumetric and cortical thickness results, we predict that adolescence-onset CD individuals will show altered microstructural integrity in the uncinate fasciculus.

### Functional imaging studies

**Figure 3:**
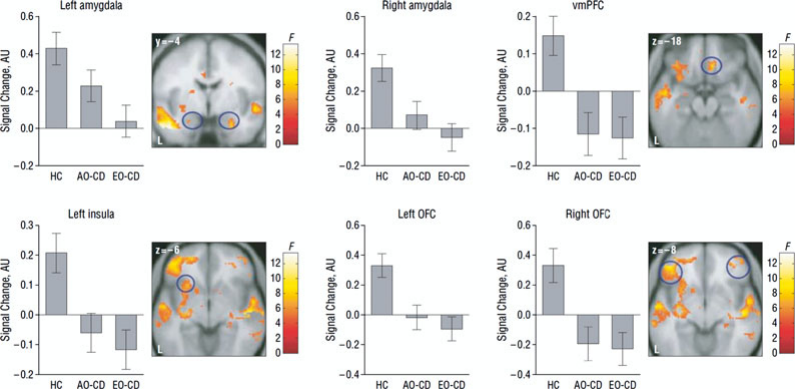
Group differences in brain activity during facial emotion processing. The graphs show neural activity in brain regions involved in emotion processing for the contrast angry minus neutral faces, split according to group status. The brain maps show regions where significant group differences in activity were observed. The early-onset conduct disorder (EO-CD; equivalent to childhood-onset CD) and adolescence-onset conduct disorder (AO-CD) groups showed reduced activity in the amygdala, ventromedial prefrontal cortex (vmPFC), insula, and orbitofrontal cortex (OFC) relative to the healthy control (HC) group (‘AU’ indicates arbitrary units). The colour bars, ranging from red to white, represent F statistics. (From Passamonti et al., 2010, *Archives of General Psychiatry*, 67, p. 733; Copyright American Medical Association, 2010.)

In addition to structural imaging studies, several studies have used functional MRI (fMRI) methods to investigate changes in brain activity in CD. In an early fMRI study, Sterzer, Stadler, Krebs, Kleinschmidt and Poustka (2005) found that male adolescents with childhood-onset CD showed abnormal anterior cingulate cortex deactivation when viewing negative images relative to controls. When controlling for anxiety symptoms, the CD group showed reduced amygdala activation relative to controls. In contrast, Herpertz et al. (2008) found increased amygdala responses to negative images relative to neutral images in adolescents with childhood-onset CD compared with controls. A study focusing on reward processing found that adolescents with childhood-onset CD showed reduced orbitofrontal cortex activation to monetary rewards, relative to healthy controls or adolescents with ADHD (Rubia et al., 2009). Finally, in the first study to investigate brain activity in adolescence-onset CD, and directly compare childhood-onset and adolescence-onset subtypes, we observed abnormal neural activation in the amygdala, anterior insula and orbitofrontal cortex during the processing of facial expressions in both CD subgroups relative to healthy controls (Passamonti et al., 2010; see Figure 3). When explicitly comparing these CD subtypes, no significant differences in neural activity were observed for the contrast angry versus neutral faces. However, for sad versus neutral faces, the childhood-onset CD group showed reduced amygdala activity relative to the adolescence-onset CD and control groups. These results appear consistent with the proposal that differences between childhood-onset and adolescence-onset forms of CD are quantitative, rather than qualitative, in nature.

In summary, a number of studies have provided evidence for changes in grey matter volume, cortical thickness and folding, anatomical connectivity and brain activity in childhood-onset conduct disorder, supporting and extending one aspect of the developmental taxonomic theory. These results are consistent with the proposal that childhood-onset CD is a neurodevelopmental disorder. However, recent studies have also challenged the view that neurobiological factors play little or no role in the aetiology of adolescence-onset antisocial behaviour. To the contrary, changes in brain structure and function have been demonstrated in adolescence-onset CD, suggesting that neurodevelopmental factors may be involved in the aetiology of this form of CD.

## Theoretical implications and reformulation

We believe that the developmental taxonomic theory has been extremely valuable in helping us to understand the heterogeneity of antisocial behaviour, and elucidating the role of neuropsychological and neurobiological factors in the aetiology of severe antisocial behaviour. The hypothesis that childhood-onset CD is a neurodevelopmental disorder which often follows a life-course persistent course has now received considerable empirical support. However, the fact that a substantial proportion of childhood-onset CD individuals appear to remit in late childhood or early adolescence needs to be explained and accommodated within the theory. In addition, adolescence-onset CD appears less benign or normative than the theory would predict, and persists into adulthood in a majority of affected individuals, who are frequently as impaired as their childhood-onset/LCP CD counterparts. Finally, the adolescence-onset CD group appears to resemble the childhood-onset CD group in terms of childhood risk factors, personality traits, neuropsychological vulnerabilities and alterations in brain structure and function. These observations support a quantitative, rather than qualitative, distinction between childhood-onset and adolescence-onset CD which is not consistent with the developmental taxonomic theory and has implications for the DSM and ICD systems.

One key issue in the literature that prompts caution in interpreting the results obtained to date is the marked variation between studies in terms of experimental designs, sampling strategies and assessment methods. These factors render comparisons across studies problematic. For example, studies based on selected samples recruited from clinics, forensic settings or the youth justice system, may be more likely to include adolescence-onset CD cases who will go on to show persistent forms of antisocial behaviour. In contrast, representative, community-based samples may contain a greater proportion of true ‘adolescence-limited’ CD cases who will desist from antisocial behaviour by late adolescence or young adulthood. A good example of these sampling biases was provided by Lahey et al. (1998), who observed differences in the relationship between gender and age-of-onset of CD when comparing high-risk and community samples: individuals with adolescence-onset CD were more likely to be female in the high-risk, clinic-referred sample, whereas in a representative sample, females were equally likely to show childhood-onset or adolescence-onset forms of CD. They also found stronger associations between childhood-onset CD and parental antisocial behaviour in their clinic-referred sample than in their representative sample. These data suggest that associations between age-of-onset of CD and gender, impairment and familial risk may differ between high-risk and population-based samples. Nevertheless, we note that epidemiological studies based on birth cohort or population-based samples have still provided considerable data that challenge the developmental taxonomic theory, such as the observation that many individuals with adolescence-onset CD continue to show serious antisocial behaviour well into adulthood or the finding that both LCP and adolescence-onset forms of CD are associated with reduced cortisol secretion.

It should also be noted that Moffitt (1993) invoked the concept of becoming ‘ensnared’ by the cumulative consequences of antisocial behaviour, to explain why some adolescence-onset individuals, who would otherwise have a good prognosis, continue to show antisocial behaviour into adulthood. According to Moffitt, becoming ensnared would include gaining a criminal record, becoming a teenage mother or developing a substance use disorder. However, we contend that ‘ensnarement’ is a difficult concept to operationalise, and the diagnostic criteria for CD require the affected individual to be impaired as a result of their disorder (American Psychiatric Association, 2000), so it could be argued that anyone with a CD diagnosis has become ensnared. The findings from the Dunedin study showing that adolescence-onset conduct problems frequently persist until at least age 26 and are associated with elevated rates of substance use disorders at age 32 (e.g., Odgers et al., 2007, 2008), suggest that becoming ‘ensnared’ is the rule rather than the exception, even in population-based or birth cohort samples.

We end our review by proposing a reformulation of the developmental taxonomic theory, which attempts to explain why individuals with adolescence-onset CD show a delayed onset to their antisocial behaviour, despite possessing similar neuropsychological and neural vulnerabilities to the childhood-onset CD group. Specifically, if neuropsychological and neurobiological deficits are present in both groups, and it is assumed that these impairments play a causal role in the emergence of antisocial behaviour, why does one group develop problems early in life while the other is relatively protected until adolescence?

**Figure 4:**
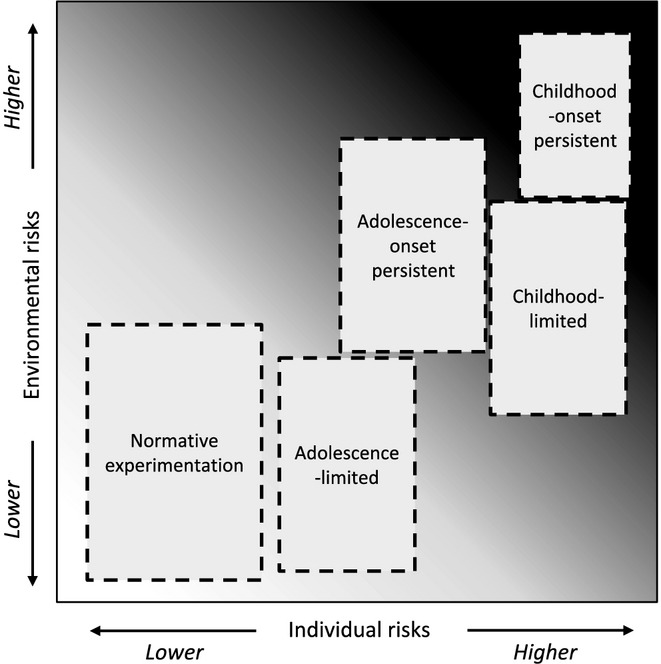
Revised developmental taxonomy for the emergence, desistence and persistence of conduct disorder across childhood and adolescence. The model depicts five clusters of children or adolescents within a matrix of individual and environmental vulnerabilities for conduct disorder. It is the combined effects of these vulnerability dimensions, and their nature and timing over the first two decades of life, that result in differential probabilistic risks for conduct disorder and the likelihood of persistence or desistence over time. The model illustrates our argument that the differences between childhood-onset and adolescence-onset conduct disorder are quantitative, rather than qualitative, in nature (although childhood-onset forms tend to be more severe and persistent, and may require a higher loading of environmental or individual-level risk factors). As the childhood-onset persistent and childhood-limited conduct disorder subtypes appear to share many risk factors in common, we propose that individual-level risk factors are high in both subtypes. Note that those in the ‘normative experimentation’ cluster would *not* merit a diagnosis of conduct disorder. Although we have been informed by the empirical literature, the dashed lines around the clusters indicate that the available evidence is too limited to confirm their exact position in the two-dimensional matrix. The boxes also show where the highest concentration of individuals in each group is expected to occur, but they are not necessarily restricted to these regions of the matrix

We propose that childhood experiences and the child's rearing environment *moderate* the temporal impact of these individual vulnerabilities. Children who are brought up in severely suboptimal conditions of poverty, poor parental supervision and childhood maltreatment (i.e., exposure to multiple, correlated environmental risk factors and a lack of psychosocial protective factors) are more likely to develop CD early in life than those whose early environment is relatively benign or even positive. Similarly, individuals who grow up in supportive families that meet their needs, and who experience warm and consistent parenting, may be relatively protected from developing antisocial behaviour given the same underlying neurobiological or personality vulnerabilities, at least until they reach adolescence and are less closely supervised and can seek out their own environments or social groups. However, if the individual possesses multiple intraindividual risk factors, this may negate the effects of protective environmental factors such as warm parenting. We also note that it is plausible that preexisting vulnerabilities in neuropsychological or brain function could be amplified by exposure to childhood adversity, domestic violence, maltreatment, or neglect, consistent with the child-family transactions that Moffitt (1993) described. Finally, negative environmental experiences may lead to intraindividual or neurobiological vulnerabilities through their effects on brain development or gene expression (i.e., epigenetic mechanisms). These proposals are consistent with classic diathesis-stress theories, but allow for the possibility that positive environments can exert protective effects (rather than just being characterised by an absence of negative experiences). Our model also suggests that early adversity can affect the *diathesis* itself (e.g., by inhibiting gene expression or disrupting brain development). In addition to a gradient of environmental risk and protective factors, we contend that individual vulnerabilities for CD are also likely to fall on a continuum (see Figure 4). Individuals with severe neuropsychological or neurological impairments may show an earlier onset of CD or more severe forms of the condition, irrespective of environmental influences. Conversely, individuals who possess relatively few individual risk factors may only develop CD if they are exposed to high levels of childhood adversity or trauma (Weder et al., 2009). Finally, these individual or environmental risks are viewed as operating dynamically, rather than being static – for example, atypical brain maturational processes occurring during adolescence or changes in the quality of the environment may trigger adolescence-onset forms of CD, even in the relative absence of childhood risk factors.

Consistent with our argument that the quality of the early environment moderates the impact of individual vulnerabilities, thereby influencing age-of-onset of CD (although we note that the same data have been used to support the developmental taxonomic theory), Raine et al. (2005) found higher rates of abuse in the LCP and childhood-limited antisocial groups than the control or AL groups. The LCP and childhood-limited groups also had higher rates of exposure to psychosocial adversity than the control and AL groups. Moffitt and Caspi (2001) also reported increased rates of adversity or environmental risk factors in the backgrounds of LCP individuals, and Fergusson et al. (2000) showed that although all of the antisocial groups they studied had higher levels of psychosocial risk factors than controls, the LCP group was exposed to the greatest number of risk factors. As such, the timing and severity of exposure to environmental adversity appears to be related to the age-of-onset and the severity of CD, at least amongst those who possess specific latent vulnerabilities for antisocial behaviour.

## Directions for future research

First, as all neuroimaging studies on CD to date have been cross-sectional, we cannot infer that the observed neurobiological differences play a causal role in the aetiology of antisocial behaviour. For example, it is plausible that alterations in brain structure partly reflect the consequences of the lifestyle choices made by individuals with CD, such as abusing substances or sustaining head injuries in physical fights. Alternatively, given that there is a strong association between exposure to childhood maltreatment and the development of CD (Caspi et al., 2002; Widom, 1989), and psychosocial adversity may alter brain development via biological embedding processes (De Brito et al., 2013; Hart & Rubia, 2012; Hertzman, 2012), it is possible that the neurobiological differences observed in CD are a consequence of childhood maltreatment or environmental adversity. However, epidemiological studies have demonstrated that exposure to childhood adversities (particularly maladaptive family functioning) appears to act as a nonspecific risk factor for multiple forms of psychopathology in adolescence (Dunn et al., 2011) and adulthood (Green et al., 2010). As a result, the precise influence of early adversity on the development of antisocial behaviour, as opposed to internalising or psychotic disorders, remains to be determined. To address these issues, future studies should adopt prospective longitudinal designs to examine whether neural or structural abnormalities are present before the onset of antisocial behaviour, and whether they predict outcomes in a probabilistic manner. These studies would also allow the researchers to be more confident about their participants' age-of-onset of CD, as they would not have to rely on retrospective reports. Future neuroimaging studies of antisocial behaviour should also take participants' early experiences into account, to investigate whether childhood adversity explains or moderates the observed group differences.

Second, although there have been several epidemiological studies focusing on childhood-limited CD, experimental work using neuropsychological and neuroimaging methods to study this group is scarce. Further research is needed, especially as it has the potential to shed light on the protective factors which enable some individuals to desist from showing behavioural difficulties. These studies should also assess experiences of psychological therapy, as receipt of treatment may be an important factor that discriminates between childhood-limited and LCP individuals.

Finally, far less is known about the causes of antisocial behaviour in females, as the majority of neuropsychological and neuroimaging studies have been restricted to males alone and very few have contained sufficient female subjects to examine whether the relationship between CD and neuropsychological or neural abnormalities differs by sex. We also know relatively little about the causes of sex differences in antisocial behaviour, despite this being one of the most robust findings in the epidemiological literature. In particular, although some very important research has been conducted on this topic (Moffitt et al., 2001), we have only a limited understanding of why childhood-onset CD is comparatively rare amongst females, whereas there is a remarkable narrowing of the sex ratio in CD prevalence during midadolescence (Moffitt et al., 2001; Silverthorn & Frick, 1999). Consequently, further neuropsychological and neurobiological studies involving females with CD or APD are needed. This line of research may enable us to identify ways to prevent the emergence, or delay the onset, of antisocial behaviour in children who are at high risk of developing such problems.

**Key points**

1. The developmental taxonomic theory (Moffitt, 1993) proposes that life-course persistent (LCP) antisocial behaviour is a neurodevelopmental disorder, whereas adolescence-limited (AL) antisocial behaviour is transient and reflects peer processes such as mimicry of antisocial peers. We evaluated this theory in the light of recent empirical research:
2. Epidemiological studies have demonstrated that childhood predictors differentiate the LCP and AL subtypes; however, prospective longitudinal studies suggest that antisocial behaviour emerging in adolescence frequently persists into adulthood and has a negative prognosis.
3. Moreover, recent studies have demonstrated similar changes in cortisol reactivity, neuropsychological performance, and brain structure and function in childhood-onset and adolescence-onset forms of conduct disorder.
4. Consistent with the developmental taxonomic theory, these studies support the view that LCP or childhood-onset antisocial behaviour is a neurodevelopmental disorder. However, they challenge the theory by suggesting that AL/adolescence-onset antisocial behaviour may also have neurodevelopmental origins. We conclude that the theory requires reformulation, which would also have implications for our diagnostic and classification systems.
